# Self-Regulation of Breathing as an Adjunctive Treatment of Insomnia

**DOI:** 10.3389/fpsyt.2018.00780

**Published:** 2019-01-29

**Authors:** Ravinder Jerath, Connor Beveridge, Vernon A. Barnes

**Affiliations:** ^1^Charitable Medical Healthcare Foundation, Augusta, GA, United States; ^2^Department of Pediatrics, Georgia Prevention Institute, Augusta University, Augusta, GA, United States

**Keywords:** insomnia, autonomic nervous system, hyper-arousal, evolutionary mismatch hypothesis, paced breathing, slow breathing, cardiorespiratory synchronization

## Abstract

Sleep is a quiescent behavioral state during which complex homeostatic functions essential to health and well-being occur. Insomnia is a very common psychiatric disorder leading to a myriad of detrimental effects including loss of concentration, memory, and performance as well as disease. Current pharmaceutical treatments can be expensive, impairing, unhealthy, and habit-forming. Relaxation techniques, such as meditation target the brain and body in contrast to pharmaceutical interventions which solely target neurotransmitter systems in the brain. In this article we present a viewpoint on the treatment of insomnia that techniques of slow, deep breathing (0.1 Hz) in adjunct to sleep hygiene and relaxation therapies may be highly effective in initiating sleep as well as facilitating falling back asleep. The autonomic nervous system is integral to sleep initiation, maintenance, and disruption. Understanding the relationship between the autonomic nervous system and sleep physiology along with the nature of sleep itself remains a challenge to modern science. We present this perspective in light of a prevailing “dysevolution” theory on the pathology of insomnia that proposes hyper-arousal characterized in part by chronic sympathetic hyperactivation and/or parasympathetic hypoactivation disrupts normal sleep onset latency, sleep quality, and sleep duration. We additionally discuss physiological mechanisms responsible for the effectiveness of the breathing treatment we describe. A better understanding of these mechanisms and autonomic pathologies of insomnia may provide support for the effectiveness of such techniques and provide relief to sufferers of this health epidemic.

## Introduction

Sleep is an essential behavioral and neurological state characterized by an absence of response to external stimuli due to temporary periods of unconsciousness along with distinct electroencephalography (EEG) changes (1, 2). Although some physiological roles of sleep have been proposed, the definitive function of sleep remains a mystery (3). The average adult needs seven or more hours of sleep per night to maintain optimal health and well-being (4). Adults who consistently get <7 h per night are more likely to be obese (5), physically inactive, active smokers, have heart disease (6), asthma, depression, diabetes, and other illnesses (7). More than a third of American adults get <7 h of sleep on a regular basis, resulting in sleep deprivation being considered a public health epidemic (8). Globally, 35% to 50% of the adult population exhibit some insomnia symptoms (9). Our culture of sleep-deprivation is in part provoked by light bulbs, computers, and hectic schedules giving us need and opportunity to stay awake in an ever-more restless world. Objective sleep measure and physiological findings have revealed that many cases of insomnia are a result of a disorder of hyper-arousal present during the day and nighttime (10–13) resulting in physiologic activation (14). In this perspective article, we support a prevailing model of insomnia, the hyper-arousal model, to describe the pathology of insomnia focusing on the autonomic component of this hyper-arousal and how it may be attenuated.

It is estimated that 109,000 vehicular crashes resulting in injury and 6,400 crashes resulting in a fatality occur in the U.S. every year which involve a drowsy driver (15). Sleep deprivation has been reported to cost the U.S. $411 billion every year through decreases in productivity and absence from work, not including medical and other costs (16). Clearly, there are vast health and economic losses that may be rectified with the right approaches. Around 4% of Americans take prescription hypnotic sleep aids, such as Z-drugs and benzodiazepines (17). These drugs can be habit forming and can disturb sleep patterns and sleep architecture (18, 19). Other adverse effects include increased risk of injury (20), withdrawal, rebound insomnia (21), suppressed respiration, increased risk of death, depression, traffic collisions, infection, and possibly cancer (22). Our perspective is that reversing disturbed sleep duration and quality induced by the stresses and technologies (23) of modern life with breathing in adjunct to relaxation and sleep-hygiene techniques rather than inundating the brain with hypnotic drugs may be a more effective long-term method to address rampant sleep-deprivation experienced world-wide.

## Evolutionary Mismatch Hypothesis

To fully understand the nature of disorders and why they become common, we must harness more information than given by the mechanistic explanations of pathophysiology and investigate the evolutionary origins of disease (24). Evolutionary mismatch hypothesis, or “dysevolution,” is a concept in evolutionary biology that describes “mismatch diseases” resulting from evolved traits that were advantageous to an organism's ancestors, but are maladaptive in the current organism due to rapid changes in environment (25–27). Such a rapid and drastic change in organism environment is evident in the dramatic environmental and lifestyle contrast between the several million years of humanoid development and the past 12,000 years of human history spurred by the agricultural and then industrial revolution (28). Many widespread maladies are suggested to be “mismatch diseases” including osteoarthritis (29, 30), cardiovascular problems, diabetes, obesity, (26), myopia, and even athlete's foot (27) Although not empirically investigated, insomnia has also been suggested to be the result of such a mismatch (27). An important aspect of the perspective asserted in this article is that such a mismatch is a key component in the pathology of modern insomnia. We support the hypothesis that a significant mismatch between the lifestyles of modern persons and our caveman and animal ancestors has led to dramatic differences in the daily operations of the autonomic nervous system (28, 31). In modern times, our frenetic nature has resulted in toxic, long-term activation of the sympathetic nervous system; a system developed by evolutionary mechanisms for short-term stress response (32, 33). We propose that this sympathetic hyperactivation and/or parasympathetic hypoactivation may significantly influence the development and maintenance of a significant fraction of modern cases of insomnia (13).

## Non-Pharmacological Interventions

Certain non-pharmacological treatments have significant support for effectiveness in managing insomnia and so we stress that slow breathing should be used in adjunct or as a component of these prevailing methods. Sleep Hygiene education and practice are often promoted as a first line of treatment for insomnia due to effectiveness (34–36), low cost, and low risk (37). It includes practices centered around homeostatic (exercising), circadian (altering light exposure), lifestyle (restricting alcohol use), and arousal factors (stress management, restricting caffeine) (38). Relaxation techniques are intended to assist in managing stress and include practices, such as meditation, muscle relaxation, and mental imagery (39). One traditional relaxation model has described the biological induction of the relaxation response as an increase in vagal tone of the autonomic nervous system (ANS) (40). Extensions of the model additionally described cognitive-behavioral components (41). Mental and physical images of forests and other natural imagery may also provoke relaxation and sympathetic inhibition (42, 43). Many meditation styles have shown to be an effective relaxation therapy for multiple components of insomnia (44–51). Due to the commonality of sleep improvements across many mediation styles, meditation may be defined generally for sleep medicine purposes as a technique utilizing calm focus of the mind along with self-induced, cognitive and muscular relaxation (52).

## Stress and the Autonomic Nervous System

Sleep is essential to complex homeostatic functions (53) regulated largely by the ANS (54). As good sleepers transition from wake to sleep, respiratory rate slows and becomes more regular as parasympathetic tone increases (54, 55). The majority of those with an ANS disorder also have a sleep disorder (56). Short sleep duration and insomnia have been associated with significantly lower levels parasympathetic activity and/or higher levels of sympathetic activity during daytime rest (57), sleep-wake transitions, and overnight sleep (58–60), characteristic of a general hyper-arousal disorder (10). Abnormalities in autonomic modulation may in part explain the elevated risk of cardiovascular disease in insomniacs (61, 62). Studies on heart rate variability and insomnia reveal increased sympathetic activity while awake before sleep and during stage-2 non-REM sleep which support the theory that autonomic hyper-arousal is a major cause of insomnia (63, 64). This state of increased nocturnal autonomic arousal in insomnia patients who are simultaneously exhausted is known in sleep clinics as “tired but wired” (56). Studies on hyper-arousal also show increased nocturnal metabolic rate, increased activation of arousal networks in the brainstem and hypothalamus, and cortical activation during sleep (65). EEG analysis has revealed that insomniacs have increased Beta and Gamma frequency activity during NREM sleep which are frequencies more associated with the waking state (66).

Unexpected and sudden stimuli can provoke the short-term stress response to protect the animal (67). Sympathetic activation is the main factor in this immediate, defensive response, stimulating the release of stress hormones which can be very harmful with long-term exposure (32). Although short-term stress may have beneficial components, such as heightened mental alertness and enhanced immunological responses (68), high levels of distress can produce a mental blockage state in which cognition is disorganized and dysfunctional (33). This state can further produce distress which in effect may prod us into a chronically distressed lifestyle (69). In addition to insomnia, long-term distress is associated with a myriad of ailments including reduced immune function (70), mental illness, cardiovascular problems, and even cancer (71). Although the immediate stress response is autonomic, secondary effects, such as those regulated by the HPA-axis occur minutes to hours after a stressor occur. Although out of the scope of this article, these secondary effects are also likely involved in promoting insomnia and so should be included in future directions for investigation into slow breathing, dysevolution, and sleep.

In pre-historic times, our “flight-or-fight” sympathetic response was typically only activated short-term in the presence of predators, enemies, or other immediate dangers (31). However, in our modern frenetic lifestyles we find ourselves in a chronically elevated sympathetic state most likely due to a combination of distress induced by our jobs, tendencies to commit to goals and responsibilities we can't achieve, and sleep deprivation (31, 33). In today's world, “The bear is always there.” A main point of this article is that this outdated survival development, which has resulted in an impaired state of “sympathoneural hypertonicity” in which our sympathetic system is regularly overactive (72), is a major promoter of sleep deprivation (13). Similar to how distress itself can produce distress, this evolutionary “mismatch disease” may create a vicious cycle where stress leads to insomnia and insomnia leads to more stress. As with other “mismatch diseases,” an effective treatment strategy will involve shifting autonomic activity patterns to more closely approximate the patterns which exist in conditions under which our species evolved (28, 29).

According to the somatic marker hypothesis and our embodied cognitive perspective, states of the mind may not only affect the body, but states of the body may affect the mind (73, 74). Thus, just as states of wakefulness and sleep influence the ANS, the ANS may influence those same states. The “somatic markers” of ANS arousal or relaxation may be recognized by the brain and produce mental arousal or relaxation (75). Norepinephrine and epinephrine are the main hormones used by the sympathetic nervous system that when released into the blood stream, act widely across the body that promote active body movement, alertness, and arousal (76). The increased circulating norepinephrine in insomniacs may inhibit sleep (58). In contrast, the effects of the parasympathetic nervous system are mediated by acetylcholine which promotes rest and relaxation (77). Our perspective is that the strong bodily arousal/relaxation induced by the ANS can stimulate mental arousal/relaxation. Thus, by promoting physiological relaxation or arousal, we assert that the state of the ANS can influence sleep. More research into how the ANS can influence mental states is needed to determine if relaxation therapies work by modulating the ANS directly or if ANS modulation is an indirect effect.

With practice of slow, deep breathing, one may attenuate this autonomic hyper-arousal and be more relaxed when it is time to sleep. Due to slow-wave sleep promoting parasympathetic tone (78), developing a regular, healthy sleep cycle would additionally stabilize the hyper-aroused autonomic tone to a calmer state. Although deep breathing is the most commonly used relaxation technique for insomnia, few studies have empirically investigated a connection between slow breathing and insomnia (79). Tsai et al. suggested that indeed autonomic dysfunction is often a part of the pathology of insomnia and reported that slow breathing can increase vagal tone resulting in improved sleep quality (13). Research is needed on the pathology of “sympathoneural hypertonicity” in insomnia, and we additionally suggest investigation into the connection between the increases in NREM beta/gamma activity in poor sleepers and autonomic dysfunction as both may play a role in mediating the relationship between insomnia and distress.

## Cardiorespiratory Synchronization

Cardiorespiratory synchronization (CS) is an interaction in which the heart rate and breathing pattern synchronize (80). In a previous article, we discussed how the CS that occurs during slow-wave sleep assists in the restorative function of sleep (81, 82). This synchronization is directly associated with an increase in parasympathetic tone observed during NREM sleep (78). Such interaction is bi-directional as CS is strongly influenced by autonomic tone (83). We support the view that voluntarily fostering CS can influence the ANS toward parasympathetic dominance (13) and may provide some restorative function needed after sleep loss (82). Meditation has been shown to foster a high degree of CS (84, 85) along with a parasympathetic response (86, 87). Stress is correlated with decreased CS and increased sympathetic tone (88). Thus, through techniques that allow for the voluntary modulation of CS, we suggest one may better manage stress.

## Slow Deep Breathing

Slow deep breathing techniques are some of the oldest and simplest techniques demonstrated to have a variety of therapeutic effects on the body and mind. The growing literature on the effects of slow breathing reveal benefits (89) for stress (90), affective mood disorder, asthma (91), and pain (92, 93), however, the literature on its effects on insomnia is minimal. Slower respiration corresponds to higher cardiorespiratory synchronization which as discussed, may promote parasympathetic tone (94). Slow, deep, regular breathing may attenuate the sympathetic component (95, 96) of hyper-arousal largely brought about by the frenetic nature of modern life (33) which may cause insomnia (10). Deeper breathing has been shown to result in stronger sympathoinhibition (97). In contrast, irregular and fast breathing have been shown to result in sympathetic excitation (98, 99). There is empirical support (13) that breathing at a frequency of 0.1 Hz is the most effective rate to combat insomnia as this rate initiates CS (100) and has been demonstrated to enhance parasympathetic activity (101). Practicing the 0.1 Hz rate before sleep was shown to improve sleep onset latency and quality in insomniacs and enhance the stability of their sleep pattern (13). Thus, we suggest 0.1 Hz as the optimal frequency for a slow breathing technique.

The efficacy of slow breathing techniques has been recognized by the military which use such techniques during combat situations to regain composure and reduce stress (102). Regular practice of a slow breathing technique overtime may provide long term correction of sympathetic over-arousal (96). In addition, slow, deep breathing has been shown to result in melatonin production which not only promotes relaxation (103), but is an essential sleep-inducing hormone (104) which promotes parasympathetic tone and inhibits sympathetic tone (105–107). Exogenous administration of melatonin can however lead to next-day drowsiness, headache, and/or dizziness (108). Melatonin levels are lower in insomniacs and much lower in long-term insomniacs, suggesting that the longer a sleep disorder exists, the more severe it gets (109). Sleep deprivation can result in increased sympathetic tone (56). This supports our view of a vicious cycle of worsening sleep deprivation (10); as sleep deprivation worsens stress, this may lead to further sympathetic hyper-arousal further worsening a sleep disorder.

Although out of the scope of this article, another mechanism by which we suggest slow deep breathing may treat insomnia has recently gained significant support, neural entrainment. Recent findings suggest that nasal respiration may act as a global organizer of neural oscillatory activity throughout the brain (110, 111). By modulating global network oscillations, respiration may exert control over cortical excitability (112). Such entrainment may provide a mechanism for breathing to alter brain waves, such as the increase in delta activity during slowed respiration (113). If respiration truly acts as a fundamental organizer of oscillatory brain activity (111, 112), then surely its modulation could be utilized to modulate brain activity to promote sleep.

## Conclusion

In this perspective article, in agreement with a prevailing evolutionary mismatch hypothesis that hyper-arousal accompanied by sympathetic hyperactivation and parasympathetic hypoactivation is a major pathogenic mechanism of insomnia, we have suggested that modulation of the ANS via slow breathing techniques in adjunct to relaxation techniques and sleep hygiene may be a more powerful tool in combating insomnia than the prevailing method of using hypnotics and other pharmaceutical interventions. In respect to the close relationship between the ANS, sleep physiology, mental state, and respiration discussed, it is clear that people have or develop the ability to alter arousal levels voluntarily. We have proposed that slow breathing and other relaxation methods may attenuate the “mismatch disease” of autonomic hyper-arousal and help people deal with the arousing pressure to sleep. Although there is significant support for the efficacy of relaxation and sleep-hygiene techniques in treating insomnia, very limited research investigating treatment with slow, deep breathing. Through our perspective, we hope to inspire debate, discussion, and future research into insomnia as a “mismatch disease” and the effectiveness of slow breathing in attenuating autonomic hyper-arousal.

## Author Contributions

Theory developed by RJ with some writing with majority of the manuscript written by CB. Editing and writing contributed by VB.

### Conflict of Interest Statement

The authors declare that the research was conducted in the absence of any commercial or financial relationships that could be construed as a potential conflict of interest.
